# Adjunctive air-polishing with erythritol in nonsurgical periodontal therapy: a randomized clinical trial

**DOI:** 10.1186/s12903-020-01363-5

**Published:** 2020-12-29

**Authors:** Holger F. R. Jentsch, Christian Flechsig, Benjamin Kette, Sigrun Eick

**Affiliations:** 1 Department of Cariology, Endodontology and Periodontology, Centre for Periodontology, University Hospital of Leipzig, Liebigstr. 12, Haus 1, 04103 Leipzig, Germany; 2 Private Dental Practice, Berlin, Germany; 3 Department of Periodontology, School of Dental Medicine, University of Bern, Bern, Switzerland

**Keywords:** Periodontitis, Subgingival instrumentation, Clinical variables, Subgingival microorganisms, Erythritol, Biomarker

## Abstract

**Background:**

This study was aimed to investigate if the adjunctive use of erythritol air-polishing powder applied with the nozzle-system during subgingival instrumentation (SI) has an effect on the outcome of non-surgical periodontal treatment in patients with moderate to severe periodontitis.

**Methods:**

Fourty-two individuals with periodontitis received nonsurgical periodontal therapy by SI without (controls, n = 21) and with adjunctive air-polishing using nozzle + erythritol powder (test, n = 21). They were analyzed for the clinical variables BOP (primary outcome at six months), probing depth (PD), attachment level, four selected microorganisms and two biomarkers at baseline, before SI as well as three and six months after SI. Statistical analysis included nonparametric tests for intra- and intergroup comparisons.

**Results:**

In both groups, the clinical variables PD, attachment level and BOP significantly improved three and six months after SI. The number of sites with PD ≥ 5 mm was significantly lower in the test group than in the control group after six months. At six months versus baseline, there were significant reductions of *Tannerella forsythia* and *Treponema denticola* counts as well as lower levels of MMP-8 in the test group.

**Conclusions:**

Subgingival instrumentation with adjunctive erythritol air-polishing powder does not reduce BOP. But it may add beneficial effects like reducing the probing depth measured as number of residual periodontal pocket with PD ≥ 5 mm when compared with subgingival instrumentation only.

**Clinical relevance:**

The adjunctive use of erythritol air-polishing powder applied with the nozzle-system during SI may improve the clinical outcome of SI and may reduce the need for periodontal surgery.

*Trial registration* The study was retrospectively registered in the German register of clinical trials, DRKS00015239 on 6th August 2018, https://www.drks.de/drks_web/navigate.do?navigationId=trial.HTML&TRIAL.

## Background

Periodontitis is not a simple bacterial infection but a complex disease where the subgingival microbiota, the immune conditions of the host and factors of the environment interact [1]. Beside the dental plaque exist other modifying factors that contribute to develop periodontal disease [2, 3]. Nevertheless the supra- and subgingival bacterial biofilm is still the substrate that is accessable for the dental practitioner. The removal of the biofilm solves the dysbiosis with the overgrowth of more virulent microorganisms in the biofilm to restart eubiosis and to reduce inflammation [4].

Subgingival instrumentation (SI) to remove the biofilm and calculus is the cornerstone to perform a causative periodontal treatment [5]. Here, the removal of the biofilm with hand and/or sonic/ultrasonic instruments has been postulated as the gold standard for the treatment of periodontitis for many years [6, 7]. Considerable improvements of bleeding on probing (BOP), probing pocket depth (PD) and clinical attachment level (AL) to avoid tooth loss due to periodontitis can be achieved [6]. Nevertheless several attempts—like additional use of lasers, photodynamic therapy and several antimicrobials—have been made to further improve the results of subgingival instrumentation.

New tools and substances to remove the biofilm from the tooth surface have been developed. Recently erythritol as an air-polishing substance has been introduced into the spectrum of treatment modalities. Subgingival air-polishing with glycine has been shown to be potentially more effective to remove the subgingival biofilm than hand and ultrasonic instruments [8]. Erythritol is a sugar alcohol (meso-1,2,3,4-Butantetrol). It is a water-soluble, chemically neutral artificial sweetener [9]. In relation to glycine, another small particle size air-polishing substance, erythritol has a smaller particle size [10]. Erythritol is suitable to remove effciently the subgingival biofilm from the root surface [10]. In addition, in vitro erythritol suppresses the formation of a dual-species biofilm of *Porphyromonas gingivalis* and *Streptococcus gordonii* via RNA and DNA depletion and metabolic changes [11]. Erythritol is also clinically appreciated due to patient’s comfort and time efficiency [12].

Subgingival instrumentation in the initial therapy needs the removal of subgingival calculus using hand and/or sonic/ultrasonic instruments. However, there are only limited data if an adjunctive use of air-polishing with erythritol is beneficial for the clinical outcome during initial subgingival instrumentation [13, 14]. Following, the aim of the present randomized clinical trial was to verify, if the adunctive use of erythritol as an air-polishing mean for the removal of the subgingival biofilm during subgingival instrumentation gives superior results in comparison to conventional SI. The hypothesis of the study was that the adjunctive use of an air-polishing device using erythritol during SI results in a significant better outcome of the SI regarding clinical, microbiological and biomarker variables in comparison with the conventional SI six months after SI.

## Methods

### Study design

The study was approved by the Ethics Commission (#AZ436/16-ek) of the Medical Faculty of the University of Leipzig. It is registered in the German register of clinical trials (DRKS00015239), and the full study protocol is deposited there. The clinical study was conducted as a randomized controlled trial with parallel design (two independent groups) in a private dental practice. The examiner as well as the laboratory personnel were blinded. Seventy-two patients were randomly selected, screened and asked to participate in the study. Fourty-nine patients (30 male and 19 female) were willing and gave their written informed consent. The clinical trial was conducted in a private dental practice (Berlin, Germany). The principles outlined in the Declaration of Helsinki, as revised in 2008, were followed to obtain the informed consents and to conduct the clinical study. Patients with moderate to severe chronic periodontitis were included in the study [15] corresponding to stage II–III, grade B of the new classification scheme for periodontal and peri-implant diseases and conditions [16].

The same experienced dentist (C. F.) performed all SIs in both study groups. All assessment of the clinical data as well as the collection of biofilm and gingival crevicular fluid (GCF) was performed by a second experienced dentist (B. K.) blinded to the study groups. The intra-examiner calibration for reliability testing resulted in κ = 0.91 for repeated measurements of PD and AL in two quadrants of eight patients. A computer-generated randomization table was used for the recruitment and blinded the randomization of 49 participants either to the test (n = 24) or control groups (n = 25) with a 1:1 allocation ratio. An assistant of the dental practice performed the assignment to interventions and the documentation. To obtain the allocation concealment a sealed opaque envelope was used. The envelope contained the treatment number of the allocation table to the specific subject. The examiner did not perform the treatment procedures and was unaware of the treatment assignment.

### Participants of the study

The inclusion criteria were age between 40 and 65 years, at least 16 natural teeth in function, periodontitis with probing depth ≤ 6 mm, at least 16 teeth with need for SI, interproximal plaque index (API) [17] ≤ 35% at the baseline appointment after two appointments of professional prophylaxis with motivation and instruction within three weeks, no diseases with influence on the periodontal disease, no diabetes mellitus, arthritis or allergies on used substances or products. Patients were excluded if they were pregnant or breastfeeding, if they were smokers with more than seven cigarettes per day, if they had a treatment with antibiotics within the six months prior to the study, need for periodontal surgery or adjunctive antibiotic treatment to the SI and if they had any periodontal treatment during the last year before the study. Based on the results of Chondros et al. [18] for BOP with a difference of 11% at baseline a minimum of 20 volunteers per group would be necessary to detect a significant difference (*p* ≤ 0.05) with a test power of 80%.

### Clinical procedures and sampling methods

In the test and control groups the clinical variables were recorded at three appointments: at baseline before SI (t1) as well as three (t2) and six months (t3) after SI. The GCF samples and the samples of subgingival biofilm were collected at the same time. The participants received full-mouth SI at sites with PD ≥ 4 mm in two sessions carried out within 24 h using hand and sonic instruments (Hu-Friedy Manufacturing Co., Chicago, IL, USA and Dentsply Sirona, Bensheim, Germany) under local anaesthesia with articaine hydrochloride/epinephrine hydrochloride (Ultracain D-S, Sanofi-Aventis, Frankfurt/Main, Germany). The criterium was a bioacceptable root surface without clinically detectable nonmineralized and mineralized material on the root surface after SI. In addition in the test group, erythritol powder (Air-Flow® Plus Powder, EMS Nyon, Switzerland) was applied for five seconds per site using the Perioflow®handpiece with the Perioflow® nozzle with the Air-Flow® Master apparatus (all EMS Nyon, Switzerland) respecting the recommendations of the manufacturer. The nozzle was introduced into the periodontal pocket as described by Hägi et al. [12]. The powder was directed perpendicular to the root surface. Via inclination of the handpiece as well as via the flexibility of the plastic nozzle device the access of interproximal buccal and oral sites was possible and the bottom of the pocket (inclusion criteria ≤ 6 mm) could be reached.

**Fig. 1:**
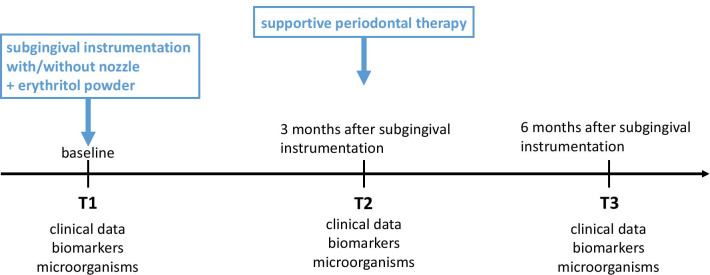
Timeline of the study using adjunctive air-polishing with erythritol in nonsurgical periodontal therapy

The use of CHX has been performed as described in the studies of Jentsch et al. [19, 20]. During the first seven days after SI all patients used a chlorhexidine digluconate mouthrinse (Chlorhexamed forte 0.2%, GlaxoSmithKline Healthcare, Bühl, Germany) for one minute twice daily. Using chlorhexidine digluconate after SI is recommended by the EFP S3 level guideline [21]. After SI careful normal oral hygiene using toothbrush and interdental brushes was performed. An appointment of comprehensive supportive periodontal therapy with removal of biofilm and calculus, (applying the erythritol powder with the Perioflow handpiece in the test group) as well as re-motivation and re-instruction took place three months after SI in both groups. In Fig. 1 the timeline of the study is presented.

In a six-point measurement per tooth (mesiobuccal, buccal, distobuccal, mesiooral, oral and distooral) the clinical variables PD, CAL and BOP of all teeth were recorded. For the assessment a manual periodontal probe (PCP-UNC 15, Hu-Friedy Manufacturing Co., Chicago, IL, U.S.A.) using a pressure of 0.25 N was used. The oral hygiene was recorded by using the API [17].

At each appointment, samples of the GCF and the subgingival biofilm were taken from the deepest site per quadrant at baseline. To sample GCF for the analysis of the biomarkers, sterile paper strips (Periopaper; Oraflow Inc., Smithtown, New York, U.S.A.) were placed at the entrance of the periodontal pocket for 30 s. As described by Griffiths [22] this intracrevicular superficial method avoids the destruction of the subgingival biofilm in the periodontal pocket. After pooling the paper strips were placed into a tube with 100 µl protease inhibitor solution (Sigma Aldrich Chemie GmbH, München, Germany). To collect the subgingival biofilm at the same sites as for GCF sampling endodontic paper points (ISO 60, Roeko GmbH, Langenau, Germany) were inserted into the pocket until resistance was felt. The paper points were left in place for 30 s. The GCF samples were stored at − 80 °C and the biofilm samples at − 20 °C until analysis.

### Laboratory analysis

Before analysis, the GCF samples were eluted overnight into 650 µl phosphate-buffered saline (+ proteinase inhibitors solution) at 4 °C. From the eluates, the levels of interleukin (IL)-1β and matrix-metalloproteinase (MMP)-8 were determined by using commercially available enzyme-linked immunosorbent assay (ELISA) kits (R & D Systems Europe Ltd., Abingdon, UK) according to the manufacturer’s instructions. The detection levels were 2 pg/site for IL-1β and 100 pg/site for MMP-8.

For microbiological analysis, DNA was extracted and a multiplex-realtime qPCR for *Aggregatibacter actinomycetemcomitans*, *Porphyromonas gingivalis*, *Tannerella forsythia* and *Treponema denticola* was performed as described recently [19]. The results are given as bacterial counts log^10^.

### Statistical analysis

The primary outcome variable of the study was BOP six months after SI. Secondary outcome variables were changes of BOP after three months as well as PD, AL, of the number of sites with PD ≥ 5 mm, counts of the four selected periodontopathogenic bacteria, the levels of IL-1β and MMP-8 three and six months after the SI.

The statistical analysis of all clinical and laboratory data was performed with the help of the software SPSS® Statistics 24.0 (IBM Corporation, New York, NY, U.S.A.). The Fisher’s exact test compared qualitative data. Non-parametric tests (Friedman-test for comparing several time-points within a group, Wilcoxon test for paired samples and Mann Whitney U-test for inter-group comparisons, respectively) without testing for normal distribution were used for intra- and inter-group comparisons. The unit of analysis in all statistical tests was the individual participant. The level of significance was α ≤ 0.05.

## Results

**Fig. 2:**
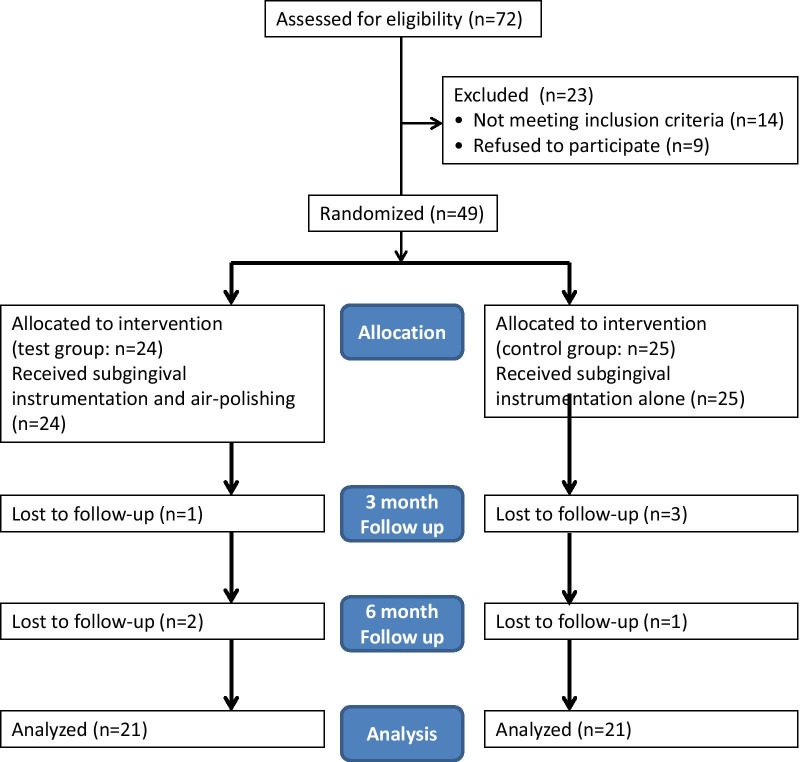
Flowchart (adapted to Moher et al. 2001) of the study using adjunctive air-polishing with erythritol in nonsurgical periodontal therapy

The study was performed from March 2017 until June 2018. In June 2018, the last patient left the study. The study flow adapted to Moher et al. [23] is presented in Fig. 2. Seventy-two patients were assessed for eligibility, 23 patients were excluded, seven patients were lost during the follow-up period. In Table 1 the demographic and baseline data of the 49 included patients are presented. There were no significant differences between both groups at baseline at the clinical variables. During the study no adverse effects of the different treatment procedures occurred and no additional medical treatment or drug intake were reported. After three months one patient was lost in the test group and three patients were lost in the control group. After six months two more patients were lost in the test group and one more patient was lost in the control group. The records of 42 participating patients with the clinical and laboratory data of all three appointments were available for statistical analysis. In Table 2 the changes of the clinical data during the study inclusive the statistical analysis are presented.

**Table 1:** Demographic data in the test (subgingival instrumentation + subgingival erythritol air-polishing) and control (subgingival instrumentation alone) groups at baseline

Variable	Test group (n = 21)	Control group (n = 21)	U-test (p)
Age (years; mean ± SD)	50.23 ± 8.26	54.29 ± 7.44	0.144
Male/female (n)	14 /7	11 /10	0.537^a^
Smoker	7	3	0.281^a^
number of teeth (mean ± SD)	25.86 ± 3.53	26.00 ± 3.08	0.949
PD (mm, mean ± SD)	3.14 ± 0.39	3.08 ± 0.36	0.697
number of PD ≥ 5 mm (mean ± SD)	29.88 ± 15.27	27.62 ± 6.54	0.667
CAL (mm, mean ± SD)	3.50 ± 0.45	3.52 ± 0.44	0.857
BOP (%, mean ± SD)	28.8 ± 31.6	27.9 ± 16.5	0.529
API (%, mean ± SD)	16.9 ± 9.7	24.3 ± 20.7	0.412

*SD* standard deviation, *n* number

^a^Fisher’s exact test, all other data Mann Whitney test

**Table 2:** Clinical variables (mean ± SD) of the test group (subgingival instrumentation + subgingival erythritol air-polishing) and control group (subgingival instrumentation alone) at baseline (T1) as well as three (T2) and six months (T3) after subgingival instrumentation incl. statistics

N_test group_ = 21 N_control group_ = 21	T1, baseline	T2, after 3 months	T3, after 6 months
Mean PD (mm)			
Test group	3.14 ± 0.39	2.44 ± 0.42***	2.23 ± 0.25***
Control group	3.08 ± 0.36	2.46 ± 0.35***	2.30 ± 0.26***
Mean CAL (mm)			
Test group	3.50 ± 0.45	2.94 ± 0.50***	2.85 ± 0.42***
Control group	3.52 ± 0.44	3.05 ± 0.44***	3.02 ± 0.45**
BOP (%)			
Test group	28.8 ± 31.6	13.5 ± 7.5**	11.5 ± 4.6**
Control group	27.9 ± 16.5	13.9 ± 9.6***	12.0 ± 9.2***
Sites PD ≥ 5 mm (n)			
Test group	29.88 ± 15.27	4.16 ± 7.68***	0.64 ± 1.38***
Control group	27.62 ± 6.54	6.54 ± 8.37***	3.12 ± 4.02***^¶¶^
API (%)			
Test group	16.9 ± 9.7	15.9 ± 17.7	16.4 ± 14.4
Control group	24.3 ± 20.7	19.7 ± 20.7	14.8 ± 9.6*

Wilcoxon’ signed rank test for paired samples: *p < 0.05, **p < 0.01, ***p < 0.001, each compared with baseline

Mann Whitney test between the groups: ^¶¶^ < 0.05

The means of PD, AL and BOP were significantly improved in both groups three and six months after SI (*p* < 0.01, *p* < 0.001) without any difference between the groups. Also the numbers of sites with PD ≥ 5 mm decreased in both groups at three months and six months (each *p* < 0.001). The number of sites with PD ≥ 5 mm was significantly lower in the test group than in the control group after six months (*p* = 0.019). Only in the control group, the oral hygiene index improved at six month, but there was no significant difference between the groups at any time-point.

**Fig. 3:**
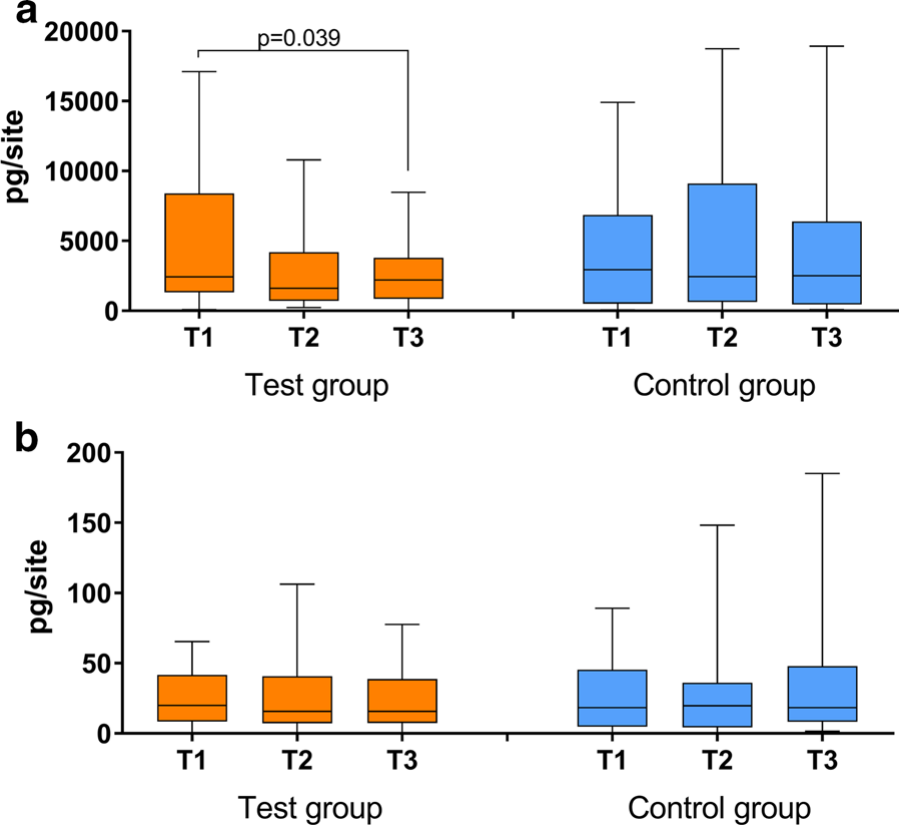
Levels of MMP-8 (**a**) and IL-1β (**b**) in gingival crevicular fluid of the test group (subgingival instrumentation + subgingival erythritol air-polishing) and control group (subgingival instrumentation alone) at baseline (T1) as well as three (T2) and six months (T3) after subgingival instrumentation incl. statistically significant results (Wilcoxon signed rank test for paired samples)

The levels of MMP-8 in the GCF decreased in the test group, they were significantly less at six months when compared with baseline, in the control group there was no change. The levels of IL-1β did not change, neither in the test nor in the control groups (Fig. 3). There was no statistical significant difference between the two groups at the studied biomarkers at any time.

**Fig. 4:**
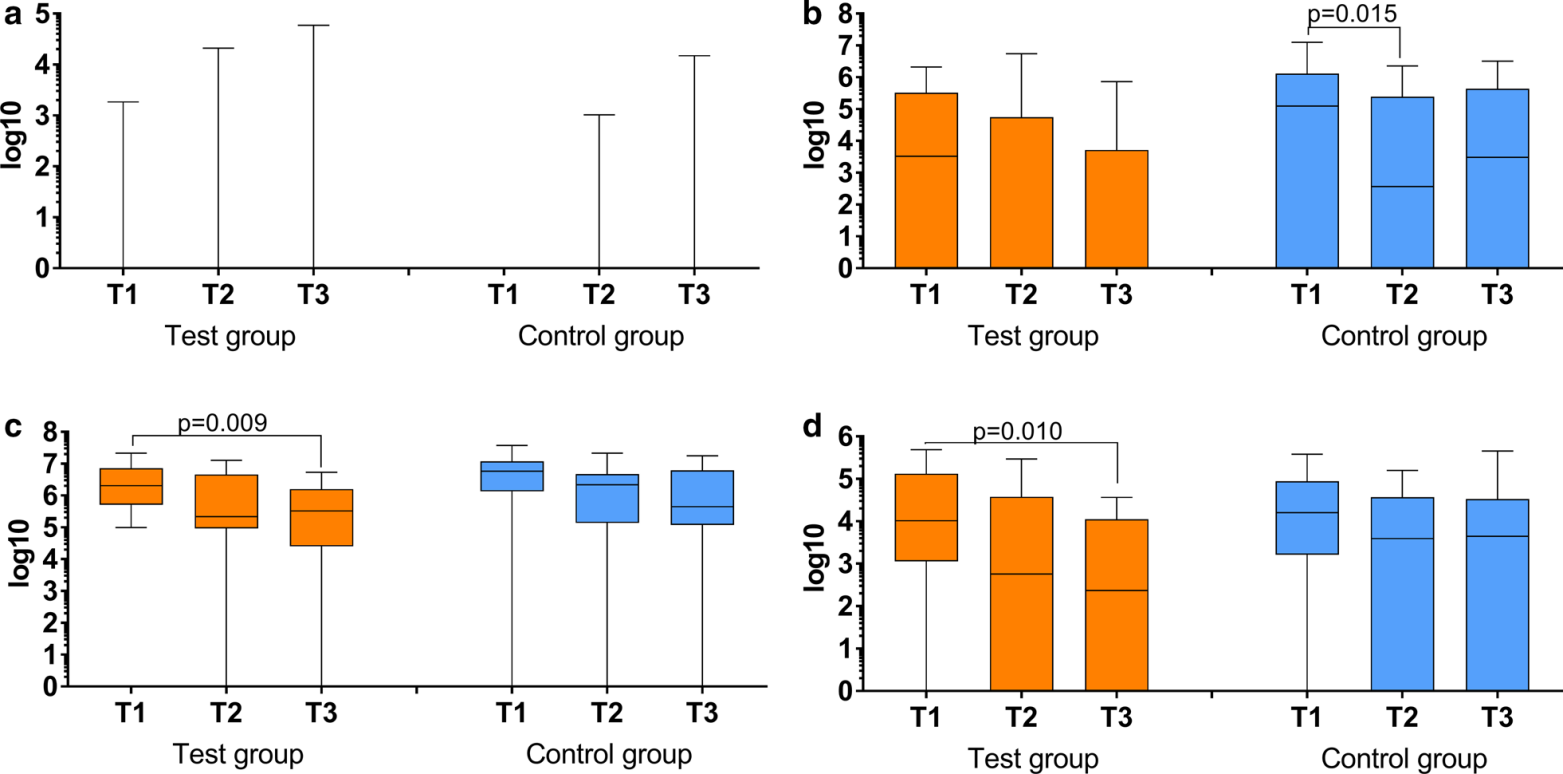
Counts of *Aggregatibacter actinomycetemcomitans* (**a**), *Porphyromonas gingivalis* (**b**), *Tannerella forsythia* (**c**) and *Treponema denticola* (**d**) in the subgingival biofilm of the test group (subgingival instrumentation + subgingival erythritol air-polishing) and control group (subgingival instrumentation alone) at baseline (T1) as well as three (T2) and six months (T3) after subgingival instrumentation incl. statistically significant results (Wilcoxon signed rank test for paired samples)

Among the selected bacteria, *A. actinomycetemcomitans* was detected only rarely. *P. gingivalis* counts were decreased in the control group at three months versus baseline. The counts of *T. forsythia* and *T. denticola* were found to be reduced at six months versus baseline in the test group (Fig. 4). There was no statistical significant difference of the counts of the selected bacteria between the two groups at any time.

## Discussion

In the present study analyzing the effect of additional air-polishing of the root surfaces with nozzle and erythritol powder during subgingival instrumentation the number of sites with PD ≥ 5 mm was significantly lower after additional air-polishing.

Reviewing the existent literature there are only limited data on the efficacy of using air-polishing with erythritol during periodontal therapy. Some studies focused on the effects of air-polishing with erythritol during the supportive periodontal therapy [10, 12, 24, 25]. Comparable results regarding BOP and PD as well as residual pockets ≥ 4 mm were reported for the nozzle use with erythritol and hand instruments, an advantage might be the low abrasiveness [10, 12]. Müller et al. [25] compared an erythritol powder supplemented with chlorhexidine and treatment with ultrasonic debridement during supportive periodontal therapy. They did not find differences in the clinical outcome after 12 months between the groups. Using a similar approach as in our study but with a split-mouth design in 21 patients and a follow-up of three months, there was also no difference between test sites and control sites [14]. In the present study, the statistical unit was the patient. Here differences were also not seen at three months but at six months. It could be assumed that also the supragingival application of air-polishing at three months was beneficial for the outcome at six months.

Glycine powder but not erythritol was applied in other studies. Wennström et al. [26] reported no significant differences of clinical variables between the use of ultrasonic instruments and air polishing with glycine powder and the nozzle device in maintenance patients during the study period of 60 days. Air-polishing with the abrasive glycine powder for 10 s per periodontal pocket as an adjunctive mean for the SI in the initial therapy of periodontitis did not lead to clinical benefits at plaque and gingival indices, probing depth, bleeding on probing and attachment level 30 days after the treatment when compared with SI only [13]. Tsang et al. [27] found no significant differences for biomarkers after adjunctive air-polishing at SI in untreated periodontitis patients during the study period of six months.

The improvement of the numbers of sites with pathological probing depths ≥ 5 mm is of clinical importance-shallow pockets without bleeding on probing are considered as pocket closure with stable periodontal conditions [28–30]. Probing depth ≥ 5 mm is an indicative to schedule the further treatment necessities because sites or teeth with residual PD ≥ 5 mm have a higher risk for further attachment loss due to disease progression as well as a higher risk for tooth loss [28, 31]. The local endpoints of periodontal therapy have been defined with shallow pockets (≤ 4 mm) without bleeding in patients with < 30% bleeding sites [32]. In our study we did not find a difference of BOP (primary outcome) between the groups. But the significant lower number of pockets with PD ≥ 5 mm six months after the combined treatment in our study suggests a reduced need for repeated SI or surgical periodontal treatment after the combination of SI and air-polishing as described for adjunctive antibiotic treatment during SI [33].

The improvement of PD in the test and control groups was in our study 0.91 and 0.78 mm, respectively. This can be considered as a medium or good improvement. Haffajee et al. [34] had an improvement of 0.45 mm for PD after six months and Jentsch et al. [20] improvements of 0.51/0.54 mm after six months.

The improvement of PD in the test group with the adjunctive use of erythritol was 0.13 mm higher than in the control group. This slightly higher improvement raises the question if it is an indication for the adjunctive use of erythritol applicated by the nozzle system. Taken into account quick and efficient therapy it is obvious that a application time of the nozzle for 5 s per site as in our study and in other studies [12, 35] instead of 10 s [13] would be more attractive for the clinician as less time for an effective treatment procedure is needed. On the other hand, the use of the nozzle as a one-way product raises the treatment costs to a certain extent. In addition, it needs some experience to introduce the device properly in the periodontal pocket.

The by 0.13 mm higher improvement in the test group is in similarity with results by other adjunctive procedures. Differences of 0.10 mm and 0.19 mm were reported for the adjunctive application of soft lasers during SI [36, 37]. Luchesi et al. [38] and Malgikar et al. [39] reported a difference of 0.07 and 0.09 mm, respectively, between the improvements of PD after SI with or without adjunctive photodynamic therapy.

At six months, in addition to the clinical variables, the level of MMP-8 was reduced in the test group. Related to air-polishing, MMP-8 level was only once determined in peri-implant sulcus fluid when applying four different cleaning procedures at dental implants after placement [40]; there was no difference to baseline and within the groups at 12 months. At six months, the counts of *T. denticola* and *T. forsythia* were also reduced only in the test group. Results reported before are different. In patients undergoing supportive therapy there was no difference between groups [12, 35, 41]. Applying air-polishing in periodontal therapy, there were reduced *P. gingivalis* counts when using air-polishing with glycine-powder supragingivally after one month [42]. The only study using air-polishing with erythritol as an adjunct, found decreased *P. gingivalis* counts at one month, but there was only a follow-up to three months [14]. Full-mouth treatment of systemically and periodontally healthy individuals with air-polishing with glycine powder revealed a significant decrease of bacteria being associated with periodontal disease up to nine days following intervention, however the counts returned to baseline after six weeks [43]. In the present study, the combination of using first subgingivally air-polishing with erythritol and after three months supragingivally may lead to the beneficial results regarding the number of sites with PD ≥ 5 mm which seemed to be associated with reduction of *T. forsythia* and *T. denticola*.

## Conclusion and limitations

The study has several limitations like e.g. no occurrence of patients-related factors and no control group with nozzle but without air abrasive powder and not well-balanced distribution of smokers between groups.

Although not showing higher improvements of BOP (primary outcome), the results of our study suggest that subgingival instrumentation with adjunctive erythritol air-polishing powder may add beneficial effects like reducing the number of residual periodontal pocket with PD ≥ 5 mm when compared with subgingival instrumentation only and may reduce the need for periodontal surgery.

## Data Availability

All data are available from the corresponding author on reasonable request.

## Abbreviations

°C: Degree celsius
AL: Clinical attachment level
API: Interproximal plaque index
BOP: Bleeding on probing
GCF: Gingival crevicular fluid
PD: Probing depth
pg: Picogram
s: Second
SI: Subgingival instrumentation
